# Fabrication, Characterization and In Vitro Assessment of *Laevistrombus canarium-*Derived Hydroxyapatite Particulate-Filled Polymer Composite for Implant Applications

**DOI:** 10.3390/polym14050872

**Published:** 2022-02-23

**Authors:** Balaji Ayyanar Chinnappan, Marimuthu Krishnaswamy, Mugilan Thanigachalam, Huaizhong Xu, Saiful Islam Khan, Md Enamul Hoque

**Affiliations:** 1 Department of Mechanical Engineering, Coimbatore Institute of Technology, Coimbatore 641014, India; kmmcit@gmail.com; 2 Department of Mechanical Engineering, Government College of Technology, Coimbatore 614013, India; mugilangct@gmail.com; 3 Department of Biobased Materials Science, Kyoto Institute of Technology (KIT), Matsugasaki Hashikamicho 1, Sakyoku, Kyoto 606-8585, Japan; 4 Department of Biomedical Engineering, Military Institute of Science and Technology (MIST), Dhaka 1216, Bangladesh; saifk28@gmail.com

**Keywords:** seashell particulates, *Laevistrombus canarium*, DSC, TGA, FESEM, cytotoxicity

## Abstract

This paper presents the formulation, characterization, and in vitro studies of polymer composite material impregnated with naturally derived hydroxyapatite (HA) particulates for biomedical implant applications. *Laevistrombus canarium* (*LC*) seashells (SS) were collected, washed and cleaned, sun-dried for 24 h, and ground into powder particulates. The SS particulates of different weight percentages (0, 10, 20, 30, 40, 50 wt%)-loaded high-density polyethylene (HDPE) composites were fabricated by compression molding for comparative in vitro assessment. A temperature-controlled compression molding technique was used with the operating pressure of 2 to 3 bars for particulate retention in the HDPE matrix during molding. The HDPE/LC composite was fabricated and characterized using X-ray diffraction (XRD), field-emission scanning electron microscopy (FESEM), energy-dispersive X-ray (EDX), differential scanning calorimetry (DSC), and TGA. Mechanical properties such as tensile, compression, flexural, hardness, and also surface roughness were tested as per ASTM standards. Mass degradation and thermal stability of the HDPE/LC composite were evaluated at different temperatures ranging from 10 to 700 °C using thermogravimetric analysis (TGA). The maximum tensile strength was found to be 27 ± 0.5 MPa for 30 wt% HDPE/LC composite. The thermal energy absorbed during endothermic processes was recorded as 71.24 J/g and the peak melting temperature (Tm) was found to be 128.4 °C for the same 30 wt% of HDPE/LC composite specimen. Excellent cell viability was observed during the in vitro biocompatibility study for EtO-sterilized 30 wt% of HDPE/LC composite specimen, except for a report of mild cytotoxicity in the case of higher concentration (50 µL) of the MG-63 cell line. The results demonstrate the potential of the fabricated composite as a suitable biomaterial for medical implant applications.

## 1. Introduction

Particulate-filled composites are used in many fast-growing industries such as automobiles, aircraft, marine, and biomedical applications due to their low density, higher specific strength, low wear rate, good corrosion resistance, biocompatibility, and biodegradability. Due to good mechanical properties, high specific strength, non-abrasive and eco-friendly properties, and cost effectiveness, they are utilized as a substitute for conventional fiber for reinforcement in composites. In recent reported studies, HAP has been widely used as a bone graft material considered for medical application due to their similar chemical composition of bone. Synthesis of HAP through a wet chemical method from waste snail shells was reported by Santosh et al. The morphology of HAP revealed the rod-shaped structure and crystallite size was about 101 nm. The XRD analysis revealed the peaks corresponding to pure HAP at the prominent planes (002), (211), (112), (202), (222), (213), and (304) [1]. The development of HAP was reported by Gergely et al. from naturally abundant eggshells. The eggshells were calcinated at 900 °C and milled for 5 h at 4000 rpm through ball milling. The functional groups were identified through the Fourier Transform Infrared (FTIR) spectrum such as carbonate, OH, and PO_4_ [2]. Kupiec et al. reported the development of HAP from porcine bones by the hydrolysis method via lactic acid, pre-calcination (600 °C), and main calcination (750–950 °C). The FTIR spectrum revealed the presence of PO_4_^3−^, OH, and CO_3_^2−^ groups in HAP derived from porcine bone [3].

HAP was also reportedly prepared by Oscar et al. from red tilapia (*Oreochromis* sp.) scales via calcination (973 °C) and acid-base treatment. The FTIR spectrum revealed the presence of OH, NH, CH, CO_3_^2−^, PO, PO_4_^3−^, and PO_4_^2–^ groups [4]. Anjaneyulu et al. reportedly synthesized nano HAP from the snail shell via the sol-gel method. The in vitro examinations of NIH-3T3 fibroblast cells that interacted with the HAP revealed a good cell interaction, cell attachment, growth of cells, and non-toxicity. The in vitro studies proved that HAP from snail shells possesses good biocompatibility (cell viability more than 90%) with no cytotoxicity [5]. Development of HAP from fish-bone (Japanese sea bream) has been reported by Ozawa et al. through heat treatment, ≤1300 °C, maintaining a porous structure, with sintered wall and a major crystalline phase. This derived HAP would be useful as inexpensive biocompatible material [6]. The development of high-purity nHAP from the eggshell was also reported by Wu et al. The shells were cleaned and crushed into powder. Then, autoclave and hydrothermal transformations were carried out at 150 °C for different reaction times. After the autoclave process, the powders were collected and dried at 60 °C for 24 h [7]. The synthesis of monetite powders from Mediterranean mussel (*Mytilus galloprovincialis*) shells is also been reportedly done by Macha et al. The powders were dried in an oven at 100 °C for 24 h and then calcinated at 800 °C for 3 h [8].

The synthesis of HAP from bovine bones (shaft portion of the bovine femurs) via the calcination method is also reported. Bovine-bone derived HAP produced with the calcination method could be an economic biomaterial when compared to other commercially accessible biomaterials [9]. The development of HAP from chicken eggshell (*Gallus domesticus*) via a hydrothermal process was reported by Oladele et al. Chicken eggshell derived HAP-filled HDPE with different percentages (10%, 20%, 30%, and 40%) were fabricated through the compression molding process. The composite with 40 wt% HAP-filled HDPE exhibited the highest flexural strength and yield strength [10]. Dhanaraj et al. reportedly extracted HAP from seashells (SS, *Anadara granosa*). The HAP powder was heated in the furnace and maintained at 900 °C for 3 h then the powder was naturally cooled. The HAP powder against pathogen bacterial strains *Escherichia coli* and *Bacillus cereus* showed excellent antibacterial activity. Hence, HAP could be more efficient and biologically important in the field of medical applications; e.g., dentine, etc. [11].

The growth of MG 63 osteoblast cells of the nHAP extracted via the enzymatic hydrolysis process from an FS tilapia (*Oreochromis* sp.) was also investigated by Huang et al. Incorporation of the FS tilapia nHAP in the matrix enhanced the mechanical properties. nHAP particles also enhanced the osteogenic differentiation and mineralization of MG-63 cells, which was confirmed by alkaline phosphate assay and Von Kossa staining. HAP was used as a biomaterial for artificial bone fabrication [12]. The extraction of HAP from tilapia scales via acid-base treatment and calcination was reportedly done by Swetha et al. The inclusion of HAP into the biopolymer matrix improves the mechanical properties. HAP is the major inorganic component of natural bone and has been used as an orthopedic, dental, and bone repair material, due to its excellent biocompatibility, osteoconductive, non-toxic, non-inflammatory, and non-immunogenic properties [13]. HAP and tricalcium phosphate (TCP) are more widely used additives, because of their close matching to natural bone units and higher mechanical properties. Nanoparticles (NPs) have increased the mechanical properties (stability, hardness, and wear-resistance) [14] and biological properties (cell proliferation, cell adhesion, and biocompatibility) of composites [15,16]. The biocompatibility of HAP derived from the *P. Jullieni* scale was studied by Pon on et al. The characterizations were revealed that the FSHAs have a large surface area, porous structure, and higher roughness which lead to increased proliferation and cell adhesion. The SBF analysis confirmed that the FSHA has a superior ability to form the apatite [17]. HAP exhibits superior biocompatibility with various kinds of cells and tissues, making it an ideal candidate for orthopedic, dental applications, and tissue engineering [18].

Many of the researchers extracted fillers from fishbone, oyster shells [19], eggshells [3,7], snail shells, bovine bones [9], and crab shells [18] have been converted into useful biomaterials. Similarly, HDPE, UHMWPE, polytetrafluoroethylene, PMMA, PLA, and PEEK are extensively used in biomedical applications, because of their excellent biocompatibility with better moldability [20,21,22]. The biocompatible composite-based feedstock filament (PLA-HAp-CS) was created using a twin-screw extruder for an open-source FDM 3D printer. The study found that 190 °C barrel temperature, 140 r/min screw speed, and 12 kg deadweight are the optimal input parameters for TSE. The optimal FDM parameters are 0.2 mm layer thickness, 30/45° deposition angle, and 100% infill density.

In situ hydroxyapatite (HAp) surface layer construction on composite ceramics (-TCP/CaSiO3) was achieved using a simple and new approach employing ultrapure water as the unique reagent for hydrothermal treatment. The surface layer is also enhanced for improved cell adhesion, and reduced cytotoxicity. An in vivo study was revealed that the manufactured biomimetic hierarchical structure scaffold would be an excellent option for bone regeneration by increasing capillary creation, bone augmentation, and new bone matrix synthesis [23].

A polyamide (PA) matrix was combined with surface-modified ZrO_2_ or Al_2_O_3_ ceramic fillers to create the composite (CFs). The powders were used to make filaments for 3D FDM printing [24,25,26]. A 3D printed PEEK composite comprising PEEK and CHAp has several biomedical uses, and its biological macromolecular behavior contributes to health sustainability because of its amazing strength and biological behavior scientific community and the medical business would benefit greatly from this comprehensive paper on 3D printing approaches for PEEK and CHAp.

The microstructure and thermal characteristics of ceramic powders formed in a ZrO_2_-CeO_2_-Y_2_O_3_-Al_2_O_3_ system are affected by the chemical composition and volume of the parent solvent. Regardless of the amount of the second oxide precursor, different morphologies of the produced powders were detected based on how much CeO_2_ precursor solution was used. As the volume of the precursor of CeO_2_ increases, the agglomerates shrink in size.

PEEK was mixed with calcium hydroxyapatite (cHAp) and reduced graphene oxide (rGO), and various lattice porous patterns were created to increase interface biocompatibility and imitate bone. The composite with the greatest rGO content of 5% has the best biocompatibility and mechanical strength. The Young’s modulus and bulk modulus of PEEK rise exponentially with the addition of rGO/cHAP from 3.85 Gpa to 54.965% with 5 wt% addition of rGO. While PEEK/rGO/cHAP composite had a greater cell aggregation and biological activity than PEEK, in vivo testing demonstrated that the NAS-DMEM composite had stronger cell growth and bioactivity [27]. HAP has been widely used in bone-tissue engineering, void fillers for orthopedics, orthopedic, and dental implant coating [28,29].

In this present research, the fillers were extracted from *L. canarium* (*Laevistrombus canarium*) seashells (SS) that are similar to the chemical composition of bones, have biodegradable properties, low density, and are plentiful in nature at a cheap cost. Seashells were chosen as fillers for the research work because they are naturally occurring, abundant in quality and free of cost. The matrix as an HDPE and fillers extracted from scale and *L. canarium* seashells were used for carrying out the present research work. This attempt to use a matrix with this combination is a novel approach and the necessary tests; i.e., the structural, mechanical characterization, thermal stability, and in vitro studies have been carried out for the development of the composite material.

## 2. Materials and Methodology

### 2.1. Materials

The filler was made from *L. canarium*, a kind of white, golden-colored seashell debris, and the SS particle density was 1.1 g/cm^3^. The HDPE employed in this study had a melting point of 125 °C, a melt flow index (MFI) of 6.0 g/10 min at 190 °C, tensile strength of 16 MPa, and 2.16 kg, and density of 0.91 g/cm^3^. The HDPE was bought from Varsha Poly Products (Coimbatore, Tamil Nadu, India).

### 2.2. Cell Line

In vitro investigations were performed using the human cell line MG-63, which was received from the National Centre for Cell Science (NCCS) in Pune, India. Cells were cultured in the institutional biotechnology laboratory using the MG-63 cell line.

### 2.3. Methodology

**Figure 1:**
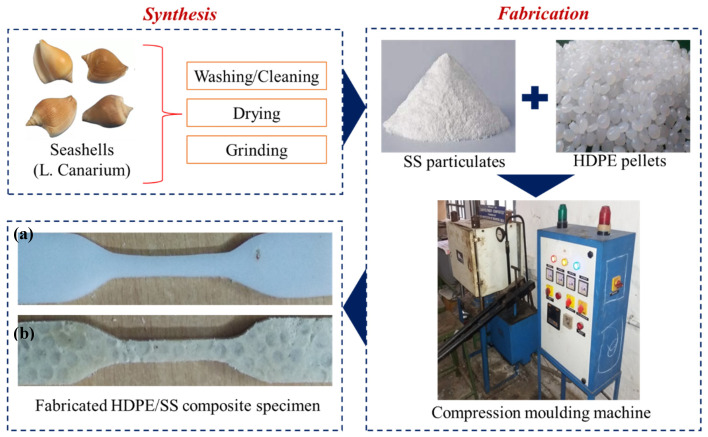
The methodology adopted to synthesize and fabricate the high-density polyethylene/seashells (HDPE/LC) dog bone-shaped composite. (**a**) Pure HDPE, 30 wt% of (**b**) SS particulate-filled HDPE composite.

To develop a new composite, a systemic approach was carried out as presented in Figure 1. The HDPE and incorporation of SS fillers with different weight percentages (0, 10, 20, 30, 40, and 50 wt.%) were used.

#### 2.3.1. Preparation of Seashell (SS) Particulates

The SS was collected from the seashore in the local area of Tirunelveli, Tamil Nādu (India). An amount of 1.5 kg irregularly sized SS was collected and washed with hot water to remove dust, and flushed. Cleaned SS were preheated under the sun for 1 week and then ground separately for 5 h at a speed of 1200 rpm to obtain fine SS particulates for further processing and analyses.

#### 2.3.2. Fabrication of Molded Composite Specimen

The pure HDPE, different wt% (0, 10, 20, 30, 40, and 50) of dry SS particulate-filled HDPE composites were fabricated through compression molding process by varying quantities of matrices and fillers. The thickness, width, length, and weight of the composites were fixed for all different wt% composites. The mass combination (in grams) of matrix (HDPE) and SS fillers for each combination of different wt% (0, 10, 20, 30, 40, and 50) of fillers in the molded composites are given in Table 1. The weight of each composite sample was fixed at 75 gms.

Different weight percentages of dry SS particulates were mixed separately with HDPE pellets and mixed manually for 5 min then kept under the compression molding machine using appropriate die. The temperature and pressure were maintained at 130 °C and 45 bar, respectively for the first 30 min, and temperature and pressure were increased to 150 °C and 100 bar, respectively, for another 30 min. In sequence, the pressure was maintained constant and the samples were allowed to attain room temperature. After reaching room temperature, the flat molded composites were removed from the dies, and further studies were carried out.

**Figure 2:**
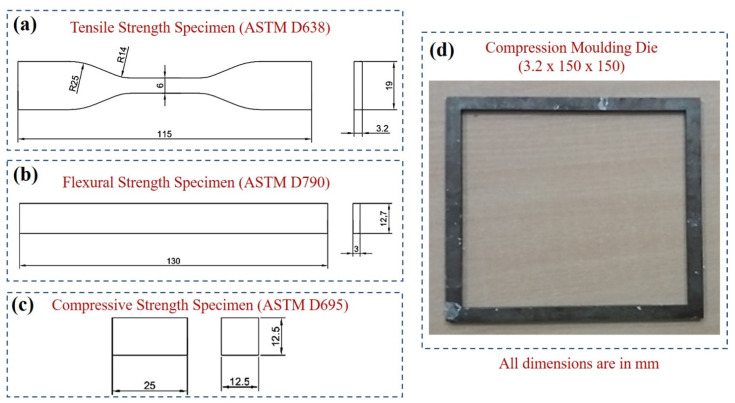
Standard specimen dimensions for mechanical testing (**a**–**d**) compression molding die (3.2 × 150 × 150 mm).

The molded flat plate composite was further prepared for a flat dog-bone-shaped specimen for carrying out the tensile strength as per ASTM D638 standards as shown in Figure 2a. The flexural strength specimen was prepared as per ASTM D790 as depicted in Figure 2b. The compressive strength specimen was prepared as per ASTM D695 as depicted in Figure 2c. The compression molding die size of 12.5 × 100 × 100 mm (thickness, width, and length) was used and is shown in Figure 2d. The specimens for hardness test (length 30 mm, and width 10) were prepared using a plastic cutter.

### 2.4. Characterization Studies

A particle size analyzer (PSA), XRD, energy-dispersive X-ray (EDX) spectroscopy, and field-emission scanning electron microscopy (FESEM) analyses of the particulates were out carried (PSGiTech, Coimbatore, India) and the results were discussed. Fourier transform infrared spectroscopy (FTIR), thermogravimetric analysis (TGA), differential scanning calorimetry (DSC), FESEM, and EDX analyses were carried out (PSGiTech, Coimbatore, India) for the 30 wt% SS particulate-filled HDPE composites and the results were discussed. Mechanical characterization was carried out using a universal tensile tester (2 mm/min) and found the tensile strength in compliance with the ASTM D638 (thickness 3 mm, and width 7 mm, span length 90 mm, and gauge length 40 mm,), compressive strength as per ASTM D695 (diameter 12.5 mm, length 25 mm), and flexural strength (thickness 3 mm, width 13 mm, and span length 80 mm) as per ASTM D790 standards. The same compositions of six HB/HDPE composite specimens were tested and the average values were reported. The Shore D hardness testing was also performed under ambient conditions on a Shore ‘D’ machine.

### 2.5. Biocompatibility Studies (Evaluation of Cytotoxicity and Cell Viability of HDPE/LC Composites)

Tests were conducted to determine the in vitro cytotoxicity of EtO-sterilized 30% of SS particulate-filled HDPE specimens using MG-63 cells. They were then allowed to interact with the MG 63 cell line in five different concentrations (10, 20, 30, 40, and 50 µL). Measurements were taken to determine the cytotoxicity and cell viability percentages. Incubating in 1× phosphate buffer saline at 37 °C for 24 h was followed by EtO sterilization of the SS particulate-filled HDPE specimens, and the replacement of MG-63 cells was undertaken using new media. At five different volumes in three sets, the SS particulate-filled HDPE liquid extract was applied to the cells. Following an 18 h incubation period at 37 °C, an MTT assay (1 mg/mL) was applied and the specimens were further incubated for 4 h. A small amount of an organic sulfur compound, dimethyl sulfoxide (DMSO), was introduced to the well plates, and readings of cytotoxicity were obtained at 570 nm on the photometer. The cell vitality and cytotoxicity were determined using the following Equations (1) and (2), respectively.

(1) $$\mathrm{Cell}\;\mathrm{vitality}=\left\{\frac{treated}{control}\right\}\times 100$$

(2) $$\mathrm{Cytotoxicity}=\left\{\frac{control-treated}{control}\right\}\times 100$$

## 3. Result and Discussions

### 3.1. FTIR Spectra of SS Particulates and SS Particulate-Filled HDPE Composite

**Figure 3:**
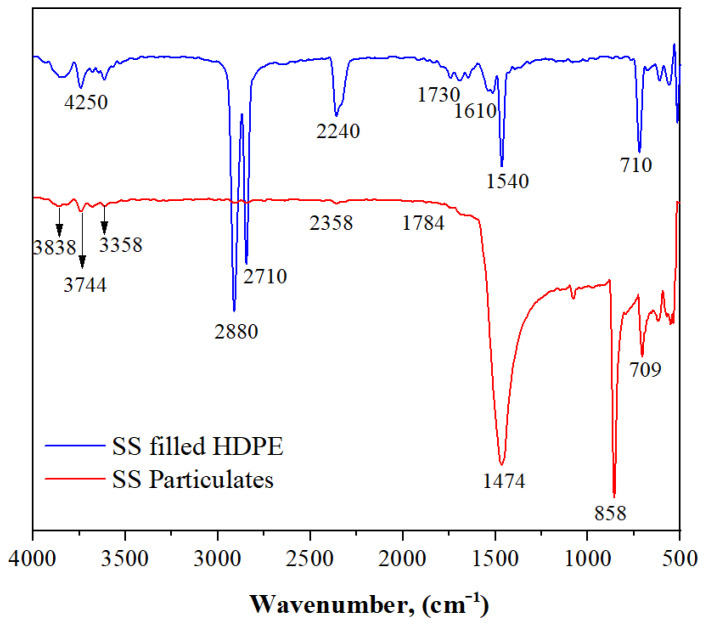
Fourier transform infrared (FTIR) spectra of SS particulate and SS particulate-filled HDPE composite.

The FTIR spectra of a seashell particulate are shown in Figure 3. The presence of the OH group is confirmed by the peak around 3838, 3744, 3678, 3612, and 3358 cm^−1^. The peak at 2356 cm^−1^ demonstrates the presence of C–C, which is predominantly found in HDPE at 1784 and 1683 cm^−1^, in the band; 89 cm^−1^ demonstrates the presence of C=O stretching vibration.

Additionally, the bands at 1474 and 1080 cm^−1^ show the existence of vibrations of groups C–C and C–O, while 858 and 709 cm^−1^ indicate the presence of various groups of C–H in SS particles. The FTIR spectrum of an SS particulate-filled HDPE composite is shown in Figure 2. In this spectrum, the typical band of about 4250 cm^−1^ was observed, which indicates the existence of the OH group. The presence of C–H can be seen in the bands about 2880 and 2710 cm^−1^, which is noticeable in HDPE. Further evidence for C–C stretching vibration is provided by the development of a band at 2240 cm^−1^. For HDPE, the bands at 1730 cm^−1^ reveal C=O vibrations, while those at 1610 cm^−1^ show N–H vibrations and those at 1540 cm^−1^ show N–O vibrations.

### 3.2. XRD of SS Particulates and 30 wt% SS Particulate-Filled HDPE Composite

**Figure 4:**
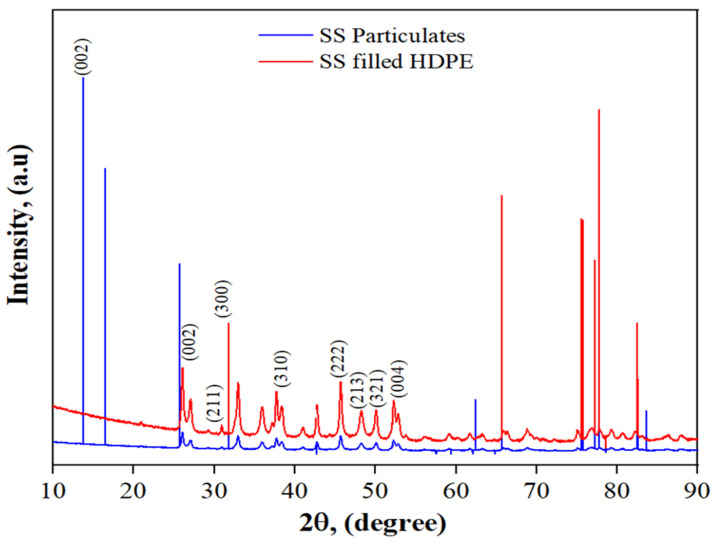
X-ray diffraction (XRD) patterns of SS particulates and 30 wt% SS particulate-filled HDPE composite.

The XRD pattern of SS particulates is shown in Figure 4. A comparison was made between the diffraction peaks and the usual XRD brag peaks obtained from hydroxyapatite (ICDD 9-432). The 2θ value for the seashell particulates was found to be 25.55, 31.15, 37.06, 41.40, 46.28, 50.13, and 53.20 corresponding to plan (002), (211), (202), (310), (222), (213), (321) and (004) which was in good agreement with the reference.

Figure 4 shows the XRD peaks of an HDPE/LC composite containing 30 wt.% SS particles that were filled with an SS powder. Compared to conventional JCPDS data, the diffraction peaks are in excellent agreement. The fact that the peaks were sharp and narrow indicated that the crystallinity was high. The 2θ value for the SS particulate-filled HDPE composite was found as 26.12, 31.02, 33.27, 39.23, 46.13, 48.88, 50.13 and 53.03 corresponding to plans (002), (211), (300), (310), (222), (213), (321) and (004). The average grain size of the SS particulates was estimated using Debye Scherer’s relation (3) over the most intense (002) peak:

(3) $$D=\frac{0.9\;\lambda \;}{\beta \;cos\;\theta }$$

where, *D* represents average grain size, *β* stands for full width at half maximum of the peak, *λ* is the diffraction wavelength (0.154059 nm) and *θ* is the diffraction [26]. The average crystallite size of the SS particulates from seashells was found to be 5.17 nm. Also, the average particle size was measured to be 2.53 ± 0.19 µm using a particle size analyzer.

### 3.3. FESEM Surface Morphology of SS Particulates and SS Particulate-Filled HDPE

**Figure 5:**
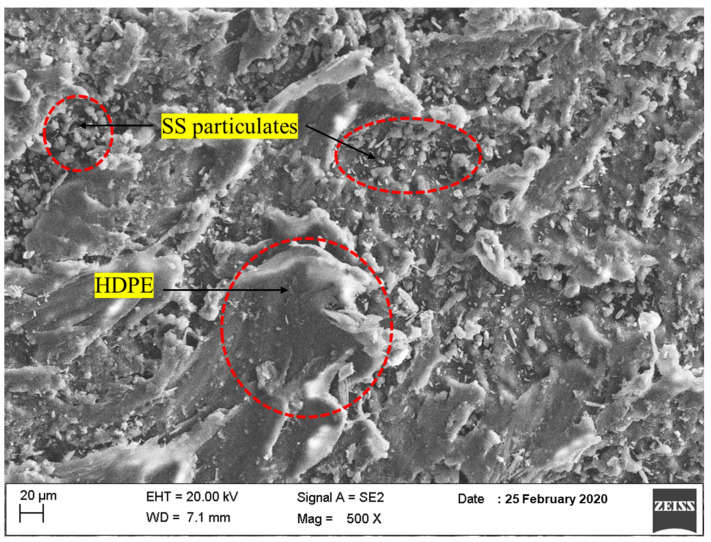
FESEM surface morphology of SS particulate-filled HDPE composite.

The morphology of SS particulates was examined using FESEM. The FESEM image of the SS particulates exhibited that the powder was having shorter and long elongated fibers in one direction and also a spherical shape, as shown in Figure 5. Also, the powder particulates have smooth and larger surface areas. The morphology of SS particulates packed in HDPE patterns demonstrates that the SS particles were evenly distributed throughout the matrix. The matrix phase is represented by grey dark background color, whereas the SS particles are represented by a silver metallic sparkling background color. The FESEM picture reveals that the particulate-blended HDPE composites agglomerated in a few areas in the specimen matrices. It is possible to manage the crater pattern by modifying the melting temperature and the right blending proportions. It was also possible to observe the orientation and plastic flow of the matrix (HDPE) and the particles.

The various elements present in the SS particulates were recognized using EDX analysis. The wt% of Ca, O, and C, were 25.07, 56.42, and 18.5 respectively. The different elements present in the SS particulate-filled HDPE composite specimens were also identified. The wt.% of C, O, and Ca were found to be 86.99, 11.27, and 1.74, respectively.

### 3.4. DSC Analysis of SS Particulate-Filled HDPE Composite

**Figure 6:**
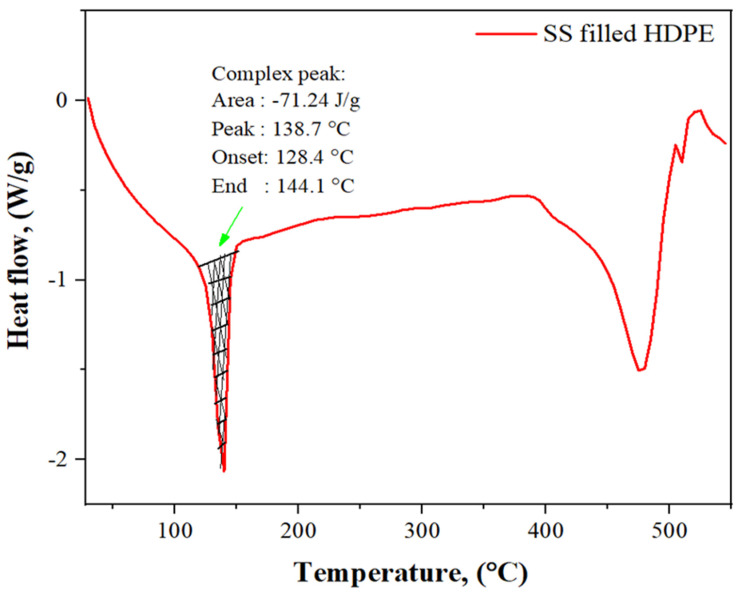
DSC curves of 30 wt% SS particulate-filled HDPE composite.

Figure 6 depicts the behavior of a 30 weight % SS (30 g) particle-filled high-density polyethylene (100 g) composite as a function of rising temperature. We identified 138.7 °C as the melting point, while 128.4 °C was found to be the melting start temperature. In the course of this endothermic reaction, it was observed that the composite had absorbed 71.24 J/g of energy. The HDPE/LC composite with 30% SS particle filling absorbs heat, which is represented by a negative peak on the graph. In this case, the polymer composite has changed from a hard, glassy solid to a softer, more elastic structure. The polymer structure can achieve sufficient flexibility by rearranging amorphous composites into crystalline forms with lower energy requirements. Last but not least, when the heat was gradually increased, the HDPE and SS powder mixture was melted to achieve the highest point of incorporation and distribution and the rapid increase in the temperature will yield an entirely amorphous polymer.

### 3.5. TGA Analysis of SS Particulate-Filled HDPE Composite

**Figure 7:**
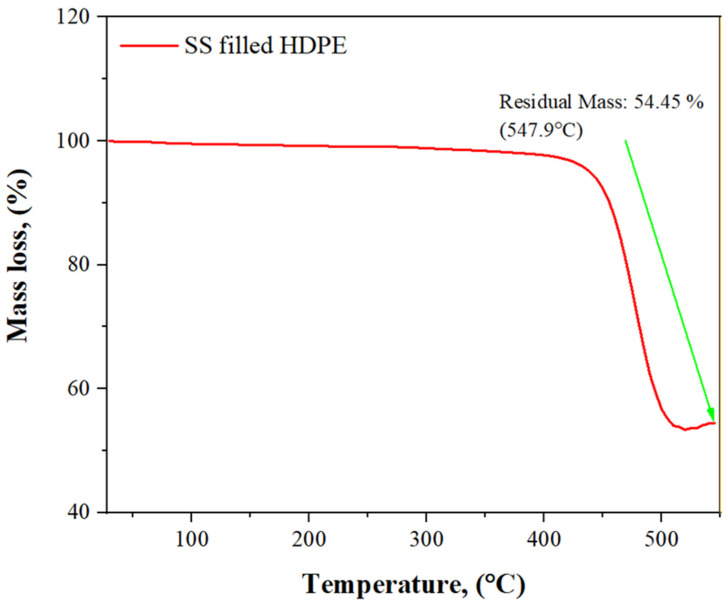
TGA curves of a 30 wt% SS particulate-filled HDPE composite.

A thermogravimetric analysis (TGA) curve in Figure 7 depicts the decrease in weight of a 30 weight % SS and SS particles-filled HDPE composite material as a function of temperature rise. As the temperature was raised gradually from 29.83 to 404.83 °C, the weight remains relatively consistent but started to decrease from 3.94 to 3.89 g. As the temperature was further raised from 504.83 to 544.83 °C, a significant loss of matrix mass occurred, from 2.17 to 2.14 mg, as increased temperature causes the components to oxidize, and eventually degrade.

## 4. Mechanical Characterization

### 4.1. Tensile Strength

The tensile strength of the HDPE/LC composite specimens with varying weight percentages (0, 10, 20, 30, 40, and 50 wt%) were tested following the ASTM 638 standard [5,30,31]. The tensile strength of the composite was gradually increased by increasing the SS particle concentrations from 0 wt% to 30 wt% over time. A progressive decrease in the tensile strength was noticed after a rise in the SS-particle concentration of more than 30 wt%, and recorded throughout the testing procedure. For the HDPE specimens without the reinforcement of the SS-particulate filler, tensile strength was found to be 24.5 ± 0.5 MPa. The maximum tensile strength of the 30 wt% of SS particulate-filled HDPE composite specimen was found to be 27. 5 ± 0.5 MPa. The tensile forces were increased by varying the particulate contents from 0 wt% up to 30 wt%, gradually. The maximum tensile force carried by the 30 wt% of SS particulate-filled HDPE specimen was found to be 425 ± 0.5 N. The force was observed to be decreased gradually when the particulate concentration was increased beyond 30 wt%. After reinforcing more than 30 wt% SS particulates in the HDPE matrix, the composite could not sustain more force due to the lack of sufficient elongation properties to carry more force. It behaves like a brittle material after adding over 30 wt% reinforcement and also spreading a greater number of particulates in the matrix.

### 4.2. Compressive Strength

The compressive strength tests were carried out for the different weight percentages (0, 10, 20, 30, 40, and 50 wt%) of SS particulate-filled HDPE specimens as per the ASTM D695 standard. The compressive strength of SS particulate-filled HDPE composite was gradually increased by increasing the natural particulates reinforcement. The results revealed that the compressive strength was seen to improve from 62 MPa to 72 MPa for the 0 wt% to 50 wt% of SS particulate-filled HDPE composite, respectively. Moreover, the compressive force was observed to be improved from 9752 N to 11165 N for the 0 wt% to 50 wt% of SS particulate-filled composite. Another reason for this was that the amount of reinforcement filler was nearly equivalent to the amount of matrix mass so that particulates were joined together, which offers poor load transferring between matrix and reinforcement.

### 4.3. Shore D Hardness

The Shore D hardness tests were carried out for the different weight percentages (0, 10, 20, 30, 40, and 50 wt%) of SS particulate-filled HDPE composite following the ASTM D2240 standard for each specimen. The Shore D hardness of the SS particulate-filled HDPE composite was observed to increase by incorporating increased natural particulates reinforcement. The D shore hardness value for specimens without SS particle reinforcement was marked at 55, while maximum shore D hardness of 67. 5 was recorded for the 50 wt% SS particulate-filled HDPE composite specimen. The indentation damage resistance of composite specimen was increased by adding a greater percentage of reinforcement filler in the HDPE.

### 4.4. Flexural Strength

The flexural test for different weight percentages (0, 10, 20, 30, 40, and 50 wt%) of SS HDPE specimens was carried out as per ASTM D790 standards for each specimen. The flexural strength was seen to increase for the composite specimens with SS fillers from 0 wt% to 30 wt% of reinforcement; however, beyond 30 wt% it was seen to decrease. The flexural strength was identified as 19.6 MPa for specimens without fillers reinforcement. On the other hand, the maximum flexural strength of the SS HDPE specimens was found to be 32 ± 0.5 MPa at 30 wt%. However, HPDE-EG (eggshell) composite with 40 wt% HA (from chicken eggshell: *Gallus domesticus*) via hydrothermal synthesis, exhibited the highest flexural and yield strength [32].

## 5. Biocompatibility Study

### 5.1. Evaluation of Cytotoxicity and Cell Viability of SS Particulate-Filled HDPE Composite

**Figure 8:**
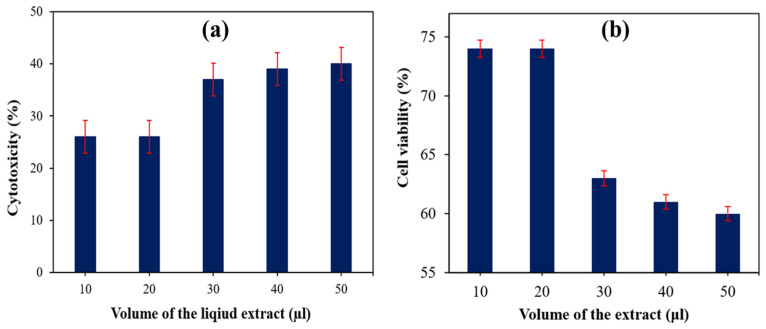
(**a**) Cytotoxicity (%) of 30 wt% of SS particulate-filled HDPE composite. (**b**) Cell viability (%) of 30 wt% SS particulate-filled HDPE composite.

Figure 8a shows the results of the direct cytotoxicity test performed on the 30 wt% SS HDPE for different volumes of the liquid extracts (10, 20, 30, 40, and 50 µL), which determine the percentage of cell viability of this composite. The results of the study are tabulated in Table 2. A comparison was made between the obtained findings with the standard reference data of cytotoxicity reactivity [33,34]. The direct cytotoxicity test was used to determine the amount of cytotoxicity (%) of 30% SS particle-filled HDPE composite specimens with five different volumes of the extract (10, 20, 30, 40, and 50 μL) allowed to contact with fresh culture media. A maximum % of cytotoxicity was found when a 50 μL liquid extract of the material interacted with new culture media, which also lies in a mild reactivity level with 60% of cell viability. Table 2 compares the amount of cytotoxicity (40%) with other levels of toxicity. The in vitro examinations of NIH-3T3 fibroblast cells that interacted with the HA revealed a good cell interaction, cell attachment, growth of cells, and non-toxicity. The in vitro studies proved that HA from snail shells has good biocompatibility (cell viability more than 90%) with no cytotoxicity [35].

Only viable cells have functioning mitochondrial dehydrogenase enzymes, which are required to convert MTT to formazan, and hence only viable cells were used in this study. SS HDPE composites with cell contacts were used to construct the composites. Cells were rinsed three times with PBS (pH 7.3) culture fluid before MTT (0.5 mg/mL) was added to cells and incubated at 37 °C for 4 h [36]. The cytotoxicity was observed as 40 wt% SS particulate-filled HDPE, which falls between grades 3 and 4 (21–50%). As the reactivity level was below a mild level, it is within an acceptable range for such situation. This result was compared with 30 wt% HAP derived from a fish scale-HDPE composite sample, which exhibited a 50–50 cell viability to cytotoxicity during the same test [36] and it is evident that HA derived from seashell also demonstrated good biocompatibility with HDPE matrix-based composites. A direct cytotoxicity test for the 30 wt% of SS particulate-filled HDPE composite specimen for different volumes of the extract (10, 20, 30, 40, and 50 μL) was performed and the results are shown in Figure 8b.

### 5.2. Cell Morphology

**Figure 9:**
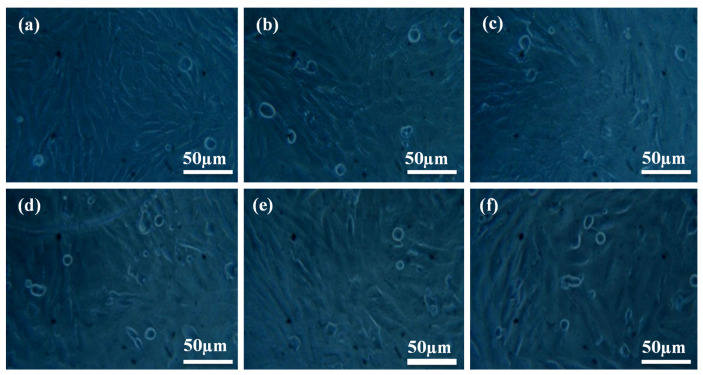
Cell morphologies of MG63 cells at (**a**) control and (**b**–**f**) after interacting with liquid extracts (10, 20, 30, 40 and 50 µL).

Figure 9a depicts the cell morphology of a fresh medium (control) 30 wt% SS-particles filled HDPE composite. The morphology of varied quantities of liquid extract given to fresh medium (10, 20, 30, 40, and 50 μL) is illustrated in Figure 9b–f. Cell viability was measured for various amounts of extract applied and compared to a control group (fresh medium) [37,38,39,40,41,42,43]. There were five different concentrations of liquid extract tested in new cell culture media to observe how they affect the cell viability. Non-living cells are represented as spherical bubbles, while live cells are shown by hairlines in the culture liquid. Cell viability was 74% and mild cytotoxicity was observed, i.e., 26% in the culture media containing 10 and 20 μL extract, respectively. Cell viability was 63% and mild cytotoxicity was 37% in the culture medium containing 30 μL. Cell viability was 61% and mild cytotoxicity was 39% in the culture medium with 40 μL. The cell viability was 60% and the mild cytotoxicity was 40% in the 50 μL culture media. Cells were observed to have numerous filopodia attaching onto the material surface after 24 h of interaction; 30 vol. % HAP-HDPE composites are bioactive and support osteoblast attachment.

## 6. Conclusions

The HAP was extracted from *Laevistrombus canarium* (*LC*) seashells (SS) that have similar chemical composition to bones. The SS particulate-filled HDPE composite was fabricated and characterized. The average particle size was measured to be of 2.53 ± 0.19 µm using a particle size analyzer. The XRD spectrum revealed the peaks of the crystal plane corresponding to the (i) seashell, and (ii) 30 wt% of SS particulate-filled HDPE composite. FTIR was used to identify the presence of both organic and inorganic polymers. The (i) SS particulate, (ii) 30 wt% of SS particulate-filled HDPE composite morphologies were investigated and elementally analyzed by EDX using FESEM. In endothermic processes, the absorbed thermal energy was found to be of 71.24 J/g and peak melt temperature (T_m_) of 128.4°C was found for 30 wt% of SS particulate-filled HDPE composite specimens, using a DSC instrument. A range of temperatures (from 10 to 700 °C) was used to test the thermal stability and mass deterioration. The 30 wt% SS particulate-filled HDPE composite exhibited the maximum tensile strength of 27 ± 0.5 MPa and thus further studies were carried out on this sample group (30 wt%). Mild cytotoxicity was identified in case of 50 μL culture medium, and excellent cell viability was observed through in vitro biocompatibility studies. The results demonstrate that the fabricated composite could be a suitable biomaterial for implant applications.

## Figures and Tables

**Table 1:** Composition of compression-molded composites (3.2 × 150 × 150 mm).

Sl. No	Different wt% of Fillers	Dry Amount of Matrix and Filler (gm)	
		HDPE	SS
1	10	67.5	7.5
2	20	60	15
3	30	52.5	22.5
4	40	45	30
5	50	37.5	37.5

**Table 2:** Cytotoxicity level of 30 wt% SS particulate-filled HDPE.

Sample Particulars		Cytotoxicity (%)	Cell Viability (%)	Cytotoxicity Reactivity
Description	The Volume of the Extract (μL)			
30 wt% SS particulate-filled HDPE	10	26	74	Mild
	20	26	74	Mild
	30	37	63	Mild
	40	39	61	Mild
	50	40	60	Mild

## Data Availability

Not applicable.
